# Measurement of the Intracochlear Hypothermia Distribution Utilizing Tympanic Cavity Hypothermic Rinsing Technique in a Cochlea Hypothermia Model

**DOI:** 10.3389/fneur.2020.620691

**Published:** 2021-01-11

**Authors:** Werner Bader, Timo Gottfried, Gerald Degenhart, Lejo Johnson Chacko, Daniel Sieber, Herbert Riechelmann, Natalie Fischer, Romed Hoermann, Rudolf Glueckert, Anneliese Schrott-Fischer, Joachim Schmutzhard

**Affiliations:** ^1^Department of Otorhinolaryngology, Medical University Innsbruck, Innsbruck, Austria; ^2^Department of Radiology, University Clinics Innsbruck, Innsbruck, Austria; ^3^MED-EL Medical Electronics GesmbH, Research and Development, Innsbruck, Austria; ^4^Department of Anatomy, Histology, and Embryology, Medical University Innsbruck, Innsbruck, Austria

**Keywords:** local hypothermia, cochlea, cochlea temperature model, surgical irrigation, temporal bone study

## Abstract

**Introduction:** Cochlea implants can cause severe trauma leading to intracochlear apoptosis, fibrosis, and eventually to loss of residual hearing. Mild hypothermia has been shown to reduce toxic or mechanical noxious effects, which can result in inflammation and subsequent hearing loss. This paper evaluates the usability of standard surgical otologic rinsing as cooling medium during cochlea implantation as a potential hearing preservation technique.

**Material and Methods:** Three human temporal bones were prepared following standard mastoidectomy and posterior tympanotomy. Applying a retrocochlear approach leaving the mastoidectomy side intact, temperature probes were placed into the basal turn (*n* = 4), the middle turn (*n* = 2), the helicotrema, and the modiolus. Temperature probe positions were visualized by microcomputed tomography (μCT) imaging and manually segmented using Amira® 7.6. Through the posterior tympanotomy, the tympanic cavity was rinsed at 37°C in the control group, at room temperature (in the range between 22 and 24°C), and at iced water conditions. Temperature changes were measured in the preheated temporal bone. In each temperature model, rinsing was done for 20 min at the pre-specified temperatures measured in 0.5-s intervals. At least five repetitions were performed. Data were statistically analyzed using pairwise *t*-tests with Bonferroni correction.

**Results:** Steady-state conditions achieved in all three different temperature ranges were compared in periods between 150 and 300 s. Temperature in the inner ear started dropping within the initial 150 s. Temperature probes placed at basal turn, the helicotrema, and middle turn detected statistically significant fall in temperature levels following body temperature rinses. Irrigation at iced conditions lead to the most significant temperature drops. The curves during all measurements remained stable with 37°C rinses.

**Conclusion:** Therapeutic hypothermia is achieved with standard surgical irrigation fluid, and temperature gradients are seen along the cochlea. Rinsing of 120 s duration results in a therapeutic local hypothermia throughout the cochlea. This otoprotective procedure can be easily realized in clinical practice.

## Background

Cochlea implantation (CI) is the gold standard procedure for patients with severe hearing loss. Hearing and structure preservation protocols have gained more and more attention over the past decade. Electrode array insertion causes direct mechanical and pathophysiological intracochlear trauma (1, 2). In rodent whole-organ culture experiments, electrode insertion trauma (EIT) has been connected with the activation of proinflammatory cytokines like interleukin (IL)-1ß, tumor necrosis factor alpha (TNF-α), cyclooxygenase-2 (COX-2), as well as enzymes like inducible nitric oxide synthase (i-NOS) (2).

Electrode insertion trauma induces both fibrosis and interstitial cell proliferation forming irregular scar tissue, resulting in loss of function (2). Otoprotective strategies inhibit inflammatory response and reduce formation of fibrotic tissue (3).

Hypothermia has been described as a potential otoprotective option (4, 5). Localized therapeutic hypothermia applied at 32–34°C inhibits intracellular inflammatory response and reactive oxygen species formation (6). Spankovich et al. demonstrated a reduction in Cisplantin-induced hearing loss. A 30°C irrigation of the external auditory canal of guinea pigs showed a significant protection from Cisplatin-induced hearing loss and outer hair cell loss (7). In gerbils, Watanabe et al. applied systemic hypothermia as otoprotective strategy to prevent hair cell loss after transient cochlear ischemia (8). In an alternative trial, KR Henry proved hypothermia to be otoprotective preventing noise-induced threshold elevations (9).

In a murine cochlea implantation trial, Tamames et al. applied a custom-made cooling device as local hypothermia source to the cochlea. On local hypothermia conditions, hearing was significantly conserved compared to the normothermic group (4).

The otoprotective effect of localized therapeutic hypothermia on the cochlea is related to hair cell protection and nerve cell preservation as shown by histological findings (4, 5). The usability of the custom-made device used in the murine experiments has been tested on human temporal bones achieving hypothermia at the round window and cochlear apex (10, 11). Additionally, a finite element model based on real-time measurements in human temporal bones was calculated (11). The simulation revealed a uniformly distributed cooling across the cochlea.

In ten temporal bone specimens, the custom-made cooling device was tested comparing myringotomy vs. a mastoidectomy approach (10). The cooling effect was documented with four microthermistors at round window, apex, lateral semicircular canals, as well as the mastoid bone. This experimental setup documented temperature distribution on two probes throughout the cochleae (10).

The access to the middle and inner ear structures during surgery requires extensive bone drilling. Drilling procedures in the human bone generate friction heat and endanger critical structures like the facial nerve, necessitating constant saline irrigation during ear surgery. Strbac et al. demonstrated in a bovine rib model significant cooling effect of saline irrigation (12). Such a cooling effect of saline irrigation in otologic hypothermia studies has not been evaluated so far.

In the current study, 12 temperature probes were positioned in the human temporal bone allowing a more detailed documentation of temperature changes. Furthermore, the use of standard surgical irrigation fluid as cooling media in the middle ear without custom-made device (4, 10) is explored.

## Methods

### Design

Human cadaver temporal bones were prepared in a two-sided approach. The external preparation consisted of standard surgical approach for cochlea implantation—mastoidectomy, antrotomy, and posterior tympanotomy. The internal preparation exposed the otic capsule and enabled the positioning of multiple temperature probes, saving the external preparations. Microcomputed tomography (μCT) scans documented the temperature probe positions. At constant 37°C, the temporal bones were rinsed with standard surgical irrigation fluid over the posterior tympanotomy for 20 min at body temperature, room temperature, and iced rinsing. Each temperature rinsing experiment was repeated at least five times. A Datalogger Graphtec GL 840 (Graphtec corporation, Japan) recorded the temperatures in 500-ms intervals.

### Datalogger Graphtec GL 840 and Temperature Probes

The datalogger GL 840 manufactured by Graphtec Corporation Japan is a processor-controlled storage unit. It records data in an adjusted rhythm. Different physical parameters can be recorded like temperature, conductivity, or pressure. GL 100_240_840-APS (Version 1.2, Graphtec Corporation, Japan) software recorded the data. The temperature probes are manufactured by Strasser GmbH & Co. KG Messtechnik (Austria). Nickel and chrome wires are welded into a small contact ball. At different temperatures, a low voltage in the millivolt range is induced.

### Preparation of Human Temporal Bone

Temporal bones were obtained from fresh cadavers donated to the Division of Clinical and Functional Anatomy. People had given their informed consent (13, 14) for their post-mortem use for scientific and educational purposes.

All cadavers had been preserved using an arterial injection of a formaldehyde–phenol solution (15).

Preparation followed a two-step procedure. Initially, a standard cochlea implantation approach was drilled. Afterwards, the cochlea and vestibulum were prepared from intracranially. The internal auditory canal (IAC) was used as anatomical landmark. The excess bone structures were reduced. This revealed the fundus visualizing the tractus spiralis foraminosus and the facial nerve canal superior to the transverse crest. The circular area of the tractus spiralis foraminosus marked the central lining of the basal turn. Then, the intact otic capsule was prepared.

**Figure 1:**
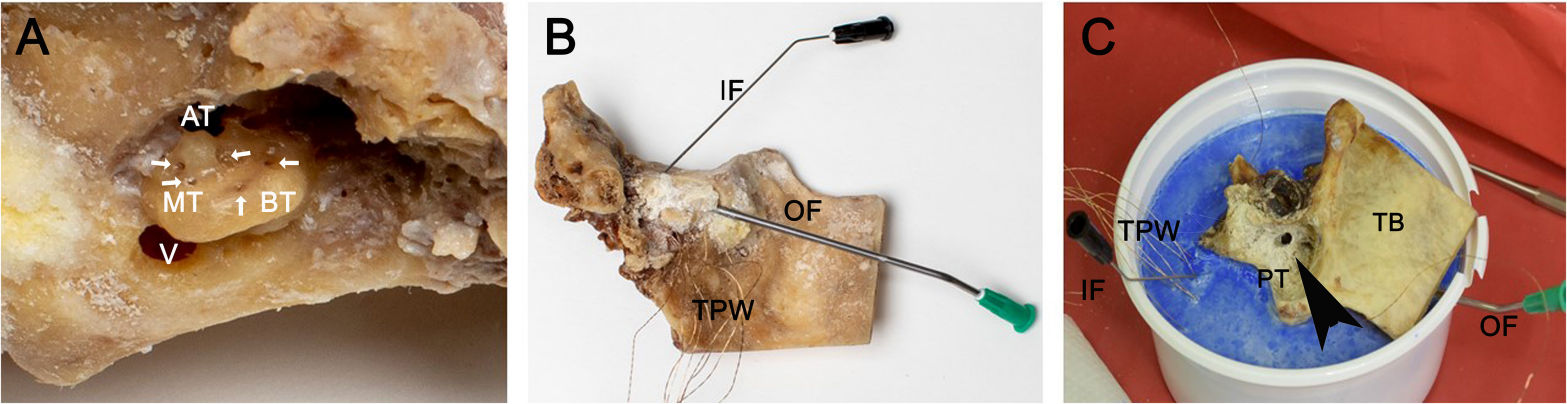
Preparation of the temporal bones. The three inserts show the basal working steps preparing the temperature model. In this example, a left temporal bone is used. **(A)** Visualizes the retrocohlear approach to the otic capsule. The large opening leads into the vestibulum. The arrows around the otic capsule mark the perforations for the temperature probe. Ap, apex; ST, second turn; BT, basal turn; V, vestibulum. **(B)** Shows the temporal bone after fixation of the temperature probes with superglue/bone dust mixture; a thin cannula measuring 0.7 mm in diameter was placed in the scala tympany posterior to the round window used as inflow. The second larger cannula with a 2-mm diameter was positioned in vestibulum for the outflow; the in- and outflows are used to rinse the cochlea to eliminate possible air bubbles. IF, inflow; OF, outflow; TPW, temperature probe wires. **(C)** The temporal bone specimen was positioned in a dental plaster socket (blue). The open mastoid with the antrotomy and posterior tympanotomy can be seen. Black arrowhead indicates the posterior tympanotomy (PT). TB, temporal bone; ZP, zygomatic process of temporal bone; TPW, temperature probe wires.

Along the facial nerve canal, the vestibulum was opened. The bony canal of the internal carotid artery was reduced, opening the anterior medial part to the otic capsule. The scala vestibuli could be identified. A 0.5-mm perforation was set at the lateral circumference of the scala vestibuli close to the scala media in each quadrant of the basal turn, each half of the second turn and the helicotrema (Figure 1A). The promontorial quadrant of the scala vestibuli was reached over the vestibule. Then, the temperature probes were positioned in predrilled openings and fixed with a superglue bone dust mixture (Figure 1B). An intracochlear rinsing construction was created to eliminate air bubbles. Therefore, a cannula (black, 0.7 mm diameter, disposable cannula Spiggle & Theis, Medizintechnik GmbH, Germany) was placed posterior to the round window. A further cannula (green, 2.0 mm diameter, disposable cannula Spiggle & Theis, Medizintechnik GmbH, Germany) was placed in the vestibule. Careful rinsing eliminated air bubbles. Then, the construction was sealed with superglue and bone dust. Careful saline rinsing confirmed impermeability. Further temperature probes were positioned at the modiolus in the tractus spiralis foraminosus and in the cavum tympany.

Finally, the cochlea model was positioned in a dental cement socket (Sockel-Plaster GT 160 by Dentona AG) containing two further diametrical temperature probes (Figure 1C). A final temperature probe was set in the rinsing tube (syringe pump infusion line, Fresenius Kabi AG) to document the rinsing temperature. Finally, 12 temperature probes were positioned as follows: two in dental cement socket, one in the rinsing tube, one in the tympanic cavity, one in the modiolus region, four in the basal turn, two in the second turn, and one in the helicotrema region.

Finishing a careful intracochlear rinse with iced water confirmed the functionality of all temperature probes recorded with the datalogger Graphtec GL 840.

### μCT Imaging

μCT technique documented the position of the temperature probes following fixation. An intracochlear 48-h conditioning with osmium tetroxide improved the contrast as previously described by Glueckert et al. (16). Then, the temporal bones were scanned at the Department of Radiology.

The μCT examinations were performed using a high-resolution peripheral quantitative CT (HR-pQCT) (XtremeCT 2 Scanco Medical AG, Brüttisellen, Switzerland). Scans were performed using 900 projections per 180° rotation with 4,609 samples, resulting in a 30.3-μm isotropic resolution. The tube settings were 68 kV voltage, 1,470 μA current, and an integration time of 900 ms per projection. Acquired images have a matrix of 4,608 × 4,608 voxels and a grayscale depth of 16 bit. The length of image stack is individually dependent and assessed using 2D scout image. Image reconstruction and DICOM processing were performed using system workstation of the μCT. DICOM data obtained by μCT scans were transferred into Amira® software (Thermo-Fisher Scientific-FEI Visualization Science Group, Méignac Cédex, France). This software enables 3D rendering, measurement, and defining. The μCT scans was segmented by hand resulting in a 3D rendering of the scala vestibuli, scala tympani, scala media, the vestibulum, and the semicircular canal. The scala media and the lateral wall were captured together to simplify the visualization. The placement of all temperature probes inside the cochlea was segmented. The 3D model visualized the temperature probe position in the cochlea model.

### Rinsing Experiments

The specimen was pre-warmed to 37°C with a heating plate. A construction of the heating plate (54°C), warming pads, and a 36–38°C water bath stabilized the temperature of the cochlea model. A perfusion pump (Becton Dickinson & Company, USA, Franklin Lakes, BD Pilot C Pump IV infusion), inflow hose, and rinsing canula (2.0 mm diameter, disposable cannula; by Spiggle & Theis, Medizintechnik GmbH) assembled the rinsing tool.

The cannula was placed in the posterior tympanotomy. The irrigation is pointed at the promontorium covering the basal turn of the cochlea. Physiological sodium chloride solution (0.9%) was used as rinse. After achieving stable temperature of 37°C in the cochlea model, rinsing was started and continued for 20 min at a flowrate of 150 ml/h. The datalogger recorded in 500-ms intervals. The rinsing was performed at 37°C, at room temperature and with iced solution. Each temperature rinse was repeated at least five times.

### Statistics

The data obtained were transferred to an Excel file. Synchronization of the data was achieved with the temperature drop of the inlet hose and the middle ear probe. The first 692 data points of the beginning to steady-state conditions and the 143 ending data points were used for further statistical analysis. The steady-state plateau data were simplified using mean values of repetitions at each measuring temperature. The included measurements were further reduced to 5-s intervals. A total of 83 values for each probe and rinsing temperature were included into the calculation. This resulted in 249 included values for every replication at each of the 12 temperature probes.

The average temperature profile of the cochleae was grouped by temperature fluid (TempFluid) and probe position (ProbePosition) and plotted graphically visualizing the steady-state conditions. Time-weighted averages for the next 150 s under steady-state conditions were calculated. These tabulated averages were grouped by ProbePosition as rows and TempFluid as columns and compared with pairwise *t*-tests at an alpha level of 0.01 with Bonferroni correction. Calculations were done using SPSS Version 26 (IBM, Armonk, NY) and MedCalc (Ostend, Belgium).

## Results

### μCT Data

**Figure 2:**
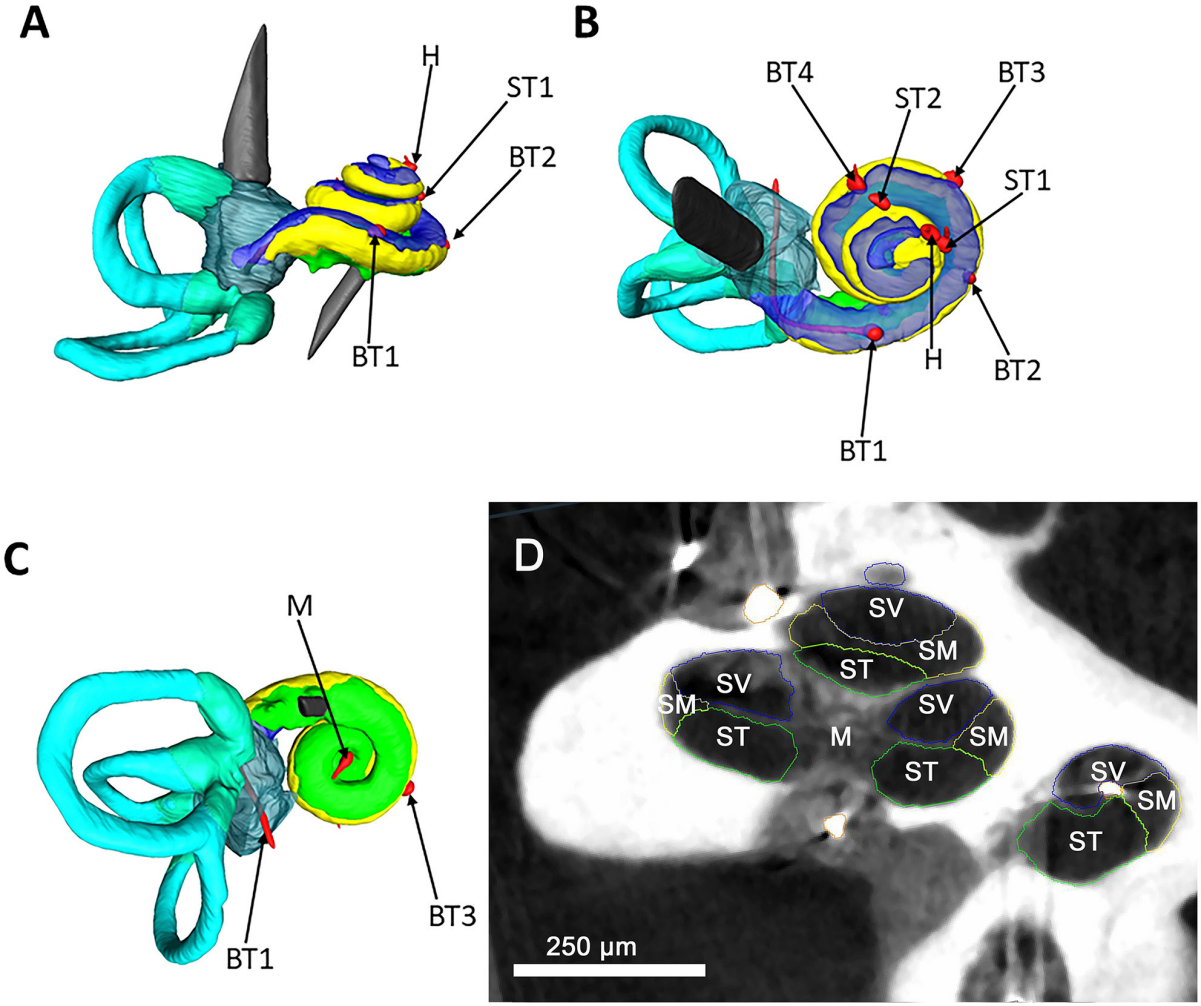
Segmented μCT data. A left cochlea and labyrinth were segmented based on μCT data; the structures are color coded as following: yellow, scala media; blue, scala vestibuli; green, scala tympani; turquoise, semicircular canals; red, temperature probes. **(A)** Represents a side view of the cochlea. A superior view is illustrated in **(B)**. **(C)** visualizes an inferior view. The red temperature probes are coded after their position as following: basal turn 1 (BT1), basal turn 2 (BT2), basal turn 3 (BT3), basal turn 4 (BT4), middle turn 1 (MT1), middle turn 2 (MT2), helicotrema (H), modiolus (M). **(D)** shows μCT image of the human cochlea and the used segmentation strategy. The main anatomical parts of the cochlea were color coded as following. Blue, scala vestibuli (SV); yellow, scala media (SM); green, scala tympani (ST), modiolus (M). For simplification of the picture, the scala media and the lateral wall were captured together as scala media. Scale bar = 250 μm.

The μCT data verified the integrity of the cochlea model. No intracochlear air enclosures were detected. The correct position of all installed temperature probes was confirmed (Figure 2).

### Temperature Rinses

Three human temporal bones were included into the study. A steady-state plateau in all temperature probes was reached after 150 and 300 s.

**Figure 3:**
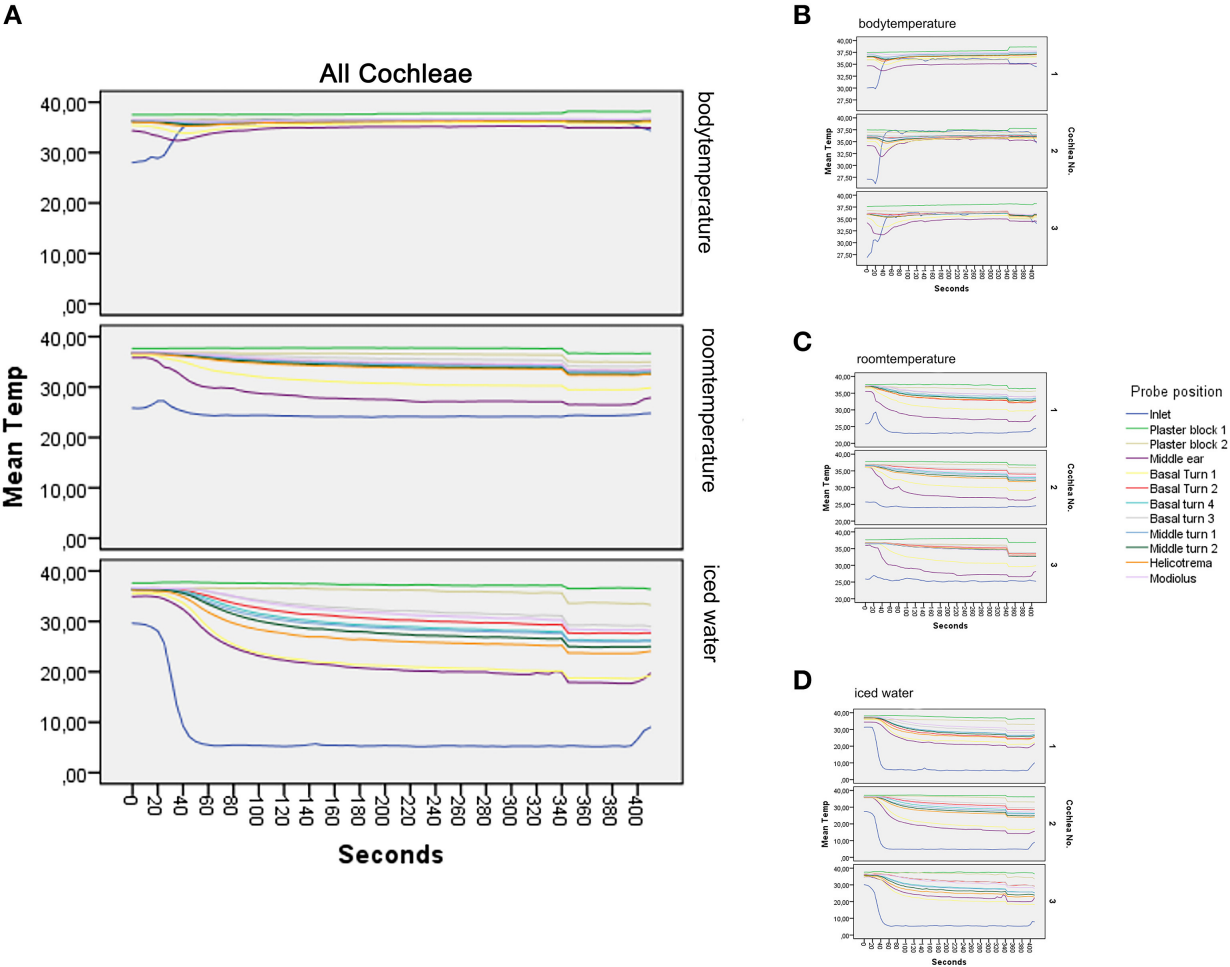
Graphical results. **(A–D)** show the main results of the experiment. **(A)** illustrates the mean value of all repetitions at the different temperatures. At the body temperature rinse, a stable level of all temperature probes at around 37°C can be seen. Rinsing at room temperature results in a deviation of all temperature curves into a mild to moderate hypothermia (17). The strongest temperature deflection was recorded at 4°C. Steady-state conditions are achieved after 150 s; detailed data of all included models are presented in **(B–D)**, with **(B)** representing the measurements at rinsing temperature of 37°C, **(C)** at room temperature, and **(D)** at 4°C. Each subgraph is the average of the included repetitions.

A total of 747 values (including three replicates) for each temperature probe were used to present the data (Figure 3).

The average rinsing temperature reached 36.52°C (SD, 0.64) at body temperature, 24.11°C (SD, 1.08) at room temperature, and 5.3°C (SD, 0.42) with iced irrigation.

#### Body Temperature Rinse (37°C)

The averaged results at 37°C are presented in Figure 3A (37°C) and the three replicates in Figure 3B. The mean values were calculated with eight repetitions for cochlea 1 and five repetitions for cochlea 2 and 3.

Starting the inlet, probe temperature increased from 29°C to a steady of 36.52°C within 40 s. A slight deflection was seen in the middle ear probe stabilizing to 35.13°C. The plaster socket and intracochlear temperatures were stable (Table 1 and Figures 3A,B).

**Table 1:** Time-weighted averages in steady state for the three cochleae by probe position (main data result).

	**TempFluid**								
	**Body temperature**			**Room temperature**			**Iced irrigation**		
**Probe position**	**Count**	**Mean**	**SD**	**Count**	**Mean**	**SD**	**Count**	**Mean**	**SD**
Inlet	3	36.52	0.64	3	24.11	1.08	3	5.30	0.42
Plaster block 1	3	37.71	0.31	3	37.75	0.27	3	37.22	0.31
Plaster block 2	3	36.37	0.45	3	36.54	0.36	3	36.11	0.59
Middle ear	3	35.13	0.33	3	27.34	0.10	3	20.33	2.85
Basal Turn 1	3	35.83	0.47	3	30.59	0.37	3	21.04	1.91
Basal Turn 2	3	36.45	0.27	3	34.66	1.36	3	30.25	3.07
Basal turn 4	3	36.39	0.42	3	34.47	0.55	3	28.88	0.75
Basal turn 3	3	36.62	0.37	3	35.57	0.58	3	32.03	1.22
Middle turn 1	3	36.40	0.68	3	34.50	0.57	3	28.51	0.39
Middle turn 2	3	36.28	0.44	3	34.05	0.72	3	27.42	0.70
Helicotrema	3	36.22	0.48	3	33.82	1.16	3	25.99	0.81
Modiolus	3	36.52	0.79	3	34.85	0.16	3	31.35	0.54

#### Room Temperature Rinse

Figure 3A visualizes the averaged results for all included measurements at 24°C rinsing.

For cochlea 1, eight repetitions were averaged and five for cochlea 2 and 3 (Figure 3C).

The inlet hose leveled at an average of 24.11°C after 30 s. The plaster block probes remained stable at ~37°C. The temperatures of the intracochlear probes dropped to hypothermic values between 30.59°C (SD, 0.37) in the basal turn 1 probe and 35.57°C (SD, 0.58) in the basal turn 3 probe (Table 1 and Figures 3A,C).

#### Iced Fluid Rinse

Figure 3A summarizes the averaged results at 5.3°C rinsing. Five repetitions for each specimen were included (Figure 3D). A sharp decrease from around 30°C to a steady-state plateau of 5.3°C (SD, 0.42) was documented within 60 s at the inlet probe. The plaster block stayed steady at 37.22°C (SD, 0.31) and 36.11 (SD, 0.59). Strong deflections were noted in the intracochlear probes—from 21.04 (SD, 1.91) in the basal turn 1 probe to 32.03°C (SD, 1.22) in the basal turn 3 probe (Table 1 and Figures 3A,D).

#### Statistical Comparison and Level of Significance

Applying a pairwise *t*-tests, the body temperature measurements were compared to rinses with room temperature and iced water. At room temperature, a statistical significance (*p* < 0.05) was noted at the following probes: basal turn 1, basal turn 4, and middle turn 2. With the iced water rinsing, a statistically significant difference (*p* < 0.05) was noted in these probes: basal turn 1, basal turn 4, basal turn 3, middle turn 1, middle turn 2, helicotrema, and modiolus.

Further, a statistical significance (*p* < 0.05) was detected at inlet probe and the middle ear probe. No difference was found in the plaster block probes (Table 2).

**Table 2:** Statistical comparison of time weighted averages in steady state for the three cochleae by sample position (main data result) for *p* < 0.05.

	**TempFluid**		
**Probe Position**	**Body temperature**	**Croom temperature**	**Iced irrigation**
	**(A)**	**(B)**	**(C)**
Inlet	B C	C	
Plaster block 1			
Plaster block 2			
Middle ear	B C	C	
Basal Turn 1	B C	C	
Basal Turn 2			
Basal turn 4	B C	C	
Basal turn 3	C	C	
Middle turn 1	C	C	
Middle turn 2	B C	C	
Helicotrema	C	C	
Modiolus	C	C	

*Results are based on two-sided tests assuming equal variances. For each significant pair, the key of the smaller category appears in the category with the larger mean. Significance level for upper case letters is (A, B, C): 0.05. Tests are adjusted for all pairwise comparisons within a row of each innermost subtable using the Bonferroni correction*.

## Discussion

Conventional surgical irrigation fluid—physiological sodium solution—was applied as cooling medium over a posterior tympanotomy. Therapeutic intracochlear hypothermia could be achieved. At room temperature (24.11°C), an intracochlear temperature deflection from an average of 30.59°C at the basal turn 1 probe to 35.57°C in the basal turn 3 probe was detected. The identical experiment with iced irrigation (5.3°C) averaged intracochlear temperatures from 21.04°C at the basal turn 1 to 32.03°C at the basal turn 3.

As a control group, body temperature rinsing was performed to exclude any external influence—like room temperature—on the measured changes. The body temperature control rinses (36.5°C) did not result in notable changes (35.8°C on basal turn 1 and 36.62°C on basal turn 3). The stable plateau in the body temperature rinses and the deflection in hypothermic attempt confirmed the experimental setup. Additionally, the constant temperature curves in the plaster block validated the local hypothermic effect on the cochlea.

Normothermia is maintained during general anesthesia with constant body temperature monitoring (18). A stable normothermic temperature distribution in the head is assumed during ear surgery. With average temperatures from 36.11 to 37.75°C in the plaster blocks, this was imitated successfully. The constant 37°C of the plaster blocks documents the temperature of the entire setup. The measured temperature decrease can be connected to the hypothermic rinsing, eliminating the cooling bias of the entire setup.

Therapeutic hypothermia is widely applied in the treatment of severe diseases like acute stroke, traumatic brain injury, or cardiac arrest (19–21). Evidence has linked the therapeutical benefit to a suppression of the release of apoptotic (21, 22) and proinflammatory molecules (23). Furthermore, a hypothermia-related reduction in the glutamate increase induced excitotoxicity (24, 25) has been described. These pathomechanisms modifiable by systemic therapeutic hypothermia have been linked to cochlea-implantation-induced trauma. In a murine organ of Corti explant model, Bas et al. showed an intracochlear upregulation of these proinflammatory and proapototic pathways (2) after electrode insertion trauma.

Localized hypothermic otoprotection during cochlea implantation has been shown by Tamames et al. (4) in a rodent cochlea implantation model. Cooling the cochlea with a fluorocarbon perfused custom-designed probe, the authors demonstrated an otoprotective effect of hypothermia (5–6°C below body temperature), significantly improving residual hearing and reducing outer hair cell loss.

This cooling approach was further evaluated in human temporal bones (10). A cooling effect of 4.5°C was recorded at the round window with different surgical approaches. An additional temperature probe was positioned at the apex, recording an average deflection of 3.38°C. Comparable hypothermic reactions were achieved with the standard surgical rinse as cooling medium, dropping at room temperature at the basal turn 1 position to 30.59°C and at the helicotrema to 33.82°C. The temperatures at these locations are comparable to prior published results (10). Interestingly, the improved spatial resolution of the cochlea temperature measurement (six additional probes) revealed an intracochlear temperature gradient, essential to consider in a future clinical trial (Figure 3). The least temperature deflection was noted in the basal turn 3 position. With just 35.57°C, the otoprotective hypothermic effect might be limited. Considering the anatomy of the cochlea in relation to the tympanic cavity, this is to be expected. The area with the least hypothermic reaction has the furthest distance to the middle ear cavity. Cooling of this anterior inferior part could be improved by pointing the irrigation axis over the promontorium, the oval window structures into the epitympanon.

At iced rinsing conditions (~5.3°C), the temperature gradient ranges from 21.4°C at the basal turn 1 to 32.03°C at the basal turn 3. The residual temperature probes are distributed between the above listed minimum and maximum deflection. A plateau phase is reached after 150 s in all positions of the cochlea. Transferring this observation into a future clinical trial, a period of 2 1/2 min of middle ear rinsing may lead to a localized hypothermic plateau in the cochlea.

A major limitation of the current experiment is the usage of human cadavers. The temperature distribution and changes in preserved human tissue differs from the living human tissue. To the best of our knowledge, the variation in temperature distribution in a living and preserved temporal bone has not been studied and published so far. This limitation has to be taken into consideration when transferring these results into a human trial. A further difference is the lack of perfusion in cadaveric bones. In the current setup, only passive warming of the cochlea has been performed by the heating plate. Due to the nature of the experiment, the cochlea blood flow could not be simulated. The cochlea is perfused by the spiral modiolar artery, a branch of the anterior inferior cerebellar artery ending in two different capillary networks (26). In vasoconstriction experiments, the diameter of the spiral modiolar artery in gerbils has been reported to average 60 μm (27). The fragility of the human vascular supply of the spiral ganglion has been further evaluated by Mei et al. (28). Considering this delicate vascular network, the influence of the blood flow to the temperature may be considered as minimal (26–28).

Additionally, the influence of the temperature probes/cannulas used on the measured temperature changes needs to be addressed. All metal parts added to the model were guided through the cement socket (Figure 1C). Prior to the rinsing, the model was pre-heated to 37°C (Figure 3). The temperature of the cement socket was constant at 37°C throughout the experiment. Assuming that the metal parts take the surrounding temperature, a hypothermic effect on the measurement can be excluded.

A further possible limitation are intracochlear air bubbles altering the local temperature distribution. This side effect was caused by the opening of the cochlea during preparation and was addressed with rinsing the cannulas, assuring a continuous fluid filling of the model (Figure 1B).

The total fluid filling of the cochlea was confirmed with the μCT images (Figure 2).

Applying middle ear rinses as cooling strategy, a question arises if the irrigation fluid itself affects the inner ear function. Saline-based surgical irrigation has been used for a long time in otology. In an *in vivo* experiment in chinchillas, saline irrigation has been used to reduce middle ear biofilms. The saline irrigation in this experiment did not affect the inner ear thresholds (29).

As shown by Tamames et al. the hypothermic stimulus has to be continued after electrode insertion (4). The irrigation of the middle ear after electrode insertion could affect the inner ear over the opening of the cochlea. A solid sealing is necessary to minimize fluid penetration. This could be achieved with sealing electrode arrays like the cork-type stopper (30).

Further applications of the otoprotective effect by hypothermic middle ear rinses can be discussed. Sudden hearing loss is connected with an acute interruption of the cochlea blood supply. In an experimental trial with gerbils, Hyodo et al. examined the cochlea function and glutamate efflux after transient cochlear ischemia. The hypothermia group recovered promptly with stable glutamate levels, whereas the normothermia group showed elevated glutamate levels combined with a functional impairment (25). In profound sudden hearing loss, explorative tympanotomy is performed in order to seal a possible perilymphatic fistula (31). This surgical approach can be utilized, if performed quickly, to apply local hypothermic middle ear rinses. Furthermore, hypothermia has been shown to protect the cochlea from noise-induced threshold elevations (9). Therefore, intraoperative local hypothermic rinses could be applied in procedures with a high risk of noise-induced hearing loss because of extensive drilling or movements on the ossicular chain.

Worldwide otologic surgical irrigation is performed at room temperature. No adverse events have been reported to the best of our knowledge. Irrigations with iced water (4°C) in the middle ear are not routinely performed in otology. The short- and long-term effects of 4°C irrigation of the middle ear mucosa for a longer time period, like 20 min, have not been examined so far. To the best of our knowledge, no clinical trial evaluating the side effects of iced middle ear rinsing has been published. Whereas, middle ear rinsing at room temperature is standard routine and can be easily implemented into clinical routine as cooling strategy, rinses with iced water need further evaluation.

Local hypothermia is an emerging therapeutic option in otology. When the irrigation solution is cooled down, it may positively affect the cochlea by reducing toxic or mechanical noxious effects (4, 7). Standard saline surgical irrigation is a possible cooling medium to the inner ear. Middle ear rinses with room temperature achieved therapeutic hypothermia ranging from 30.59 to 35.57°C. With iced irrigation, even lower intracochlear temperatures were achieved (21.04–32.03°C). This established otologic procedure used as cooling strategy could be easily transferred into daily routine. The otoprotective effect of hypothermia in humans needs further confirmation.

## Data Availability Statement

The original contributions generated for the study are included in the article/supplementary materials, further inquiries can be directed to the corresponding author.

## Ethics Statement

Ethical review and approval was not required for the study on human participants in accordance with the local legislation and institutional requirements. The patients/participants provided their written informed consent to participate in this study.

## Author Contributions

WB data measurement experiments, data recording, and assistants work on temporal bone. TG 3D segmentation of micro-CT data. GD micro-Ct scans. LJ, DS, NF, RG, and AS-F proof reading and advisory work. HR statistical evaluation in SPSS. RH provision and fixation of anatomical samples. JS proof reading, advisory word and preparation of temporal bone specimen. All authors contributed to the article and approved the submitted version.

## Conflict of Interest

DS is employed by the company MED-EL GmbH. The remaining authors declare that the research was conducted in the absence of any commercial or financial relationships that could be construed as a potential conflict of interest.
